# Role of UHRF1 in *de novo* DNA methylation in oocytes and maintenance methylation in preimplantation embryos

**DOI:** 10.1371/journal.pgen.1007042

**Published:** 2017-10-04

**Authors:** Shoji Maenohara, Motoko Unoki, Hidehiro Toh, Hiroaki Ohishi, Jafar Sharif, Haruhiko Koseki, Hiroyuki Sasaki

**Affiliations:** 1 Division of Epigenomics and Development, Medical Institute of Bioregulation, Kyushu University, Fukuoka, Japan; 2 RIKEN Center for Integrative Medical Sciences, Kanagawa, Japan; 3 AMED-CREST, Japan Agency for Medical Research and Development, Tokyo, Japan; Cornell University, UNITED STATES

## Abstract

The methylation of cytosine at CG sites in the mammalian genome is dynamically reprogrammed during gametogenesis and preimplantation development. It was previously shown that oocyte-derived DNMT1 (a maintenance methyltransferase) is essential for maintaining and propagating CG methylation at imprinting control regions in preimplantation embryos. In mammalian somatic cells, hemimethylated-CG-binding protein UHRF1 plays a critical role in maintaining CG methylation by recruiting DNMT1 to hemimethylated CG sites. However, the role of UHRF1 in oogenesis and preimplantation development is unknown. In the present study, we show that UHRF1 is mainly, but not exclusively, localized in the cytoplasm of oocytes and preimplantation embryos. However, smaller amounts of UHRF1 existed in the nucleus, consistent with the expected role in DNA methylation. We then generated oocyte-specific *Uhrf1* knockout (KO) mice and found that, although oogenesis was itself unaffected, a large proportion of the embryos derived from the KO oocytes died before reaching the blastocyst stage (a maternal effect). Whole genome bisulfite sequencing revealed that blastocysts derived from KO oocytes have a greatly reduced level of CG methylation, suggesting that maternal UHRF1 is essential for maintaining CG methylation, particularly at the imprinting control regions, in preimplantation embryos. Surprisingly, UHRF1 was also found to contribute to *de novo* CG and non-CG methylation during oocyte growth: in *Uhrf1* KO oocytes, transcriptionally-inactive regions gained less methylation, while actively transcribed regions, including the imprinting control regions, were unaffected or only slightly affected. We also found that *de novo* methylation was defective during the late stage of oocyte growth. To the best of our knowledge, this is the first study to demonstrate the role of UHRF1 in *de novo* DNA methylation *in vivo*. Our study reveals multiple functions of UHRF1 during the global epigenetic reprogramming of oocytes and early embryos.

## Author summary

The methylation of cytosine at CG sites in the mammalian genome is an epigenetic modification that is important for cell differentiation and embryonic development. During oocyte growth, the actively transcribed regions gain both CG and non-CG methylation. However, after fertilization, such methylation is globally erased, except for certain gene control regions and a subset of retrotransposons that retain CG methylation. We examined the role of UHRF1, a protein essential for the maintenance of CG methylation in somatic cells, in oocytes and preimplantation embryos by generating oocyte-specific *Uhrf1* gene knockout mice. We found that oocyte-derived maternal UHRF1 protein was important for nuclear localization of DNMT1 (a maintenance DNA methyltransferase) and for CG maintenance methylation, particularly at the imprinting control regions, in preimplantation embryos. Unexpectedly, we found that the gain in CG and non-CG methylation in oocytes was also affected by *Uhrf1* knockout in certain genomic regions. To the best of our knowledge, this is the first study to demonstrate a role of UHRF1 in *de novo* DNA methylation *in vivo*. Our study reveals multiple functions of UHRF1 during the global epigenetic reprogramming of oocytes and preimplantation embryos.

## Introduction

DNA methylation is a key epigenetic modification that is involved in various cellular processes, including cell differentiation, transposon silencing, genomic imprinting, and carcinogenesis [1,2]. This covalent epigenetic modification normally occurs at the C5 position of the cytosine (C) ring. In most mammalian cell types, the product of this modification, 5-methylcytosine (5mC), almost exclusively occurs in a CG context (CG methylation). However, non-CG methylation exists in certain cells such as oocytes [3–5], brain cells [6], and embryonic stem cells (ESCs) [7,8].

CG methylation is dynamically reprogrammed during mammalian gametogenesis and preimplantation development. In mouse germ cell development, CG methylation is first erased in primordial germ cells, partly due to the low expression of UHRF1 (Ubiquitin-like with PHD and RING finger domains 1) [9], a factor that is essential for maintenance methylation at CG sites [10,11] (see below). The hydroxylation of 5mC also plays a role in removing CG methylation through active or passive demethylation, particularly at the imprinting control regions (ICRs) [12,13], which dictate the parental-origin-specific expression of the imprinted genes in somatic cells [14]. Subsequently, in the female germline, an oocyte-specific methylation pattern is established in GOs by a *de novo* DNA methyltransferase (DNMT) complex composed of DNMT3A and DNMT3L [4,5,15–17]. *De novo* methylation continues until the fully grown oocyte (FGO) stage, with the genome of the oocyte accumulating both CG methylation and non-CG methylation [3–5]. It is known that the highly methylated regions in oocytes correspond to the actively transcribed regions [4,18,19], which are marked by histone H3 lysine 36 trimethylation (H3K36me3) [20]. Since DNMT3A recognizes H3K36me3 through its PWWP domain [21], the enzyme appears to methylate regions that are premarked with this modification. *De novo* methylation in oocytes also requires histone replacement, since the disruption of the H3.3 chaperone HIRA results in a global reduction in CG methylation [22].

After fertilization, with the exception of certain regions such as the ICRs and a subset of retrotransposons, the gamete-specific methylation patterns are globally erased [4,23]. Notably, the paternal genome undergoes 5mC hydroxylation, followed by active or passive demethylation [24–27]. Several factors, including ZFP57, TRIM28, DPPA3, and maintenance-type DNA methyltransferase DNMT1 are required for maintaining CG methylation at the ICRs [28–33] against the wave of hydroxylation-dependent and hydroxylation-independent demethylation. In a study using oocyte-specific *Dnmt1* knockout (KO) mice, we previously reported that, although DNMT1 is mainly localized in the cytoplasm, a small amount of oocyte-derived maternal DNMT1 in the cell nucleus engages in CG maintenance methylation at the ICRs in preimplantation embryos [31].

Mouse UHRF1 is a multi-domain protein that is essential for the CG maintenance methylation in proliferating somatic cells [10,11]. The conventional KO of *Uhrf1* results in an embryonic lethal phenotype, which is accompanied by global genomic hypomethylation [10]. UHRF1 contains five major protein domains: an ubiquitin-like (UBL) domain, a tandem Tudor domain (TTD), a plant homeodomain (PHD) finger, a SET and RING associated (SRA) domain, and a really interesting new gene (RING) finger [34–36]. UHRF1 recognizes hemimethylated CG sites through its SRA domain [37–39], and ubiquitinates H3K18 and H3K23 via the E3 ubiquitin ligase activity of its RING finger [40,41]. Subsequently, DNMT1 recognizes H3K18 and/or other ubiquitinated lysine residues and methylates the unmodified C of hemimethylated CG sites to copy the existing methylation patterns. In addition, the TTD and PHD finger of this protein recognize H3K9me2/me3 [35,36,42,43] and unmodified H3R2 [44,45], respectively, suggesting their roles in the proper localization of this protein.

Unlike DNMT1, however, the role of UHRF1 during oogenesis and preimplantation development has not been explored. We therefore generated oocyte-specific *Uhrf1* conditional KO mice and examined the impact on DNA methylation in oocytes and preimplantation embryos. Our study revealed an expected role of maternal UHRF1 in preimplantation embryos and an unexpected new function in GOs.

## Results

### The subcellular localization of UHRF1 in oocytes and preimplantation embryos

**Fig 1:**
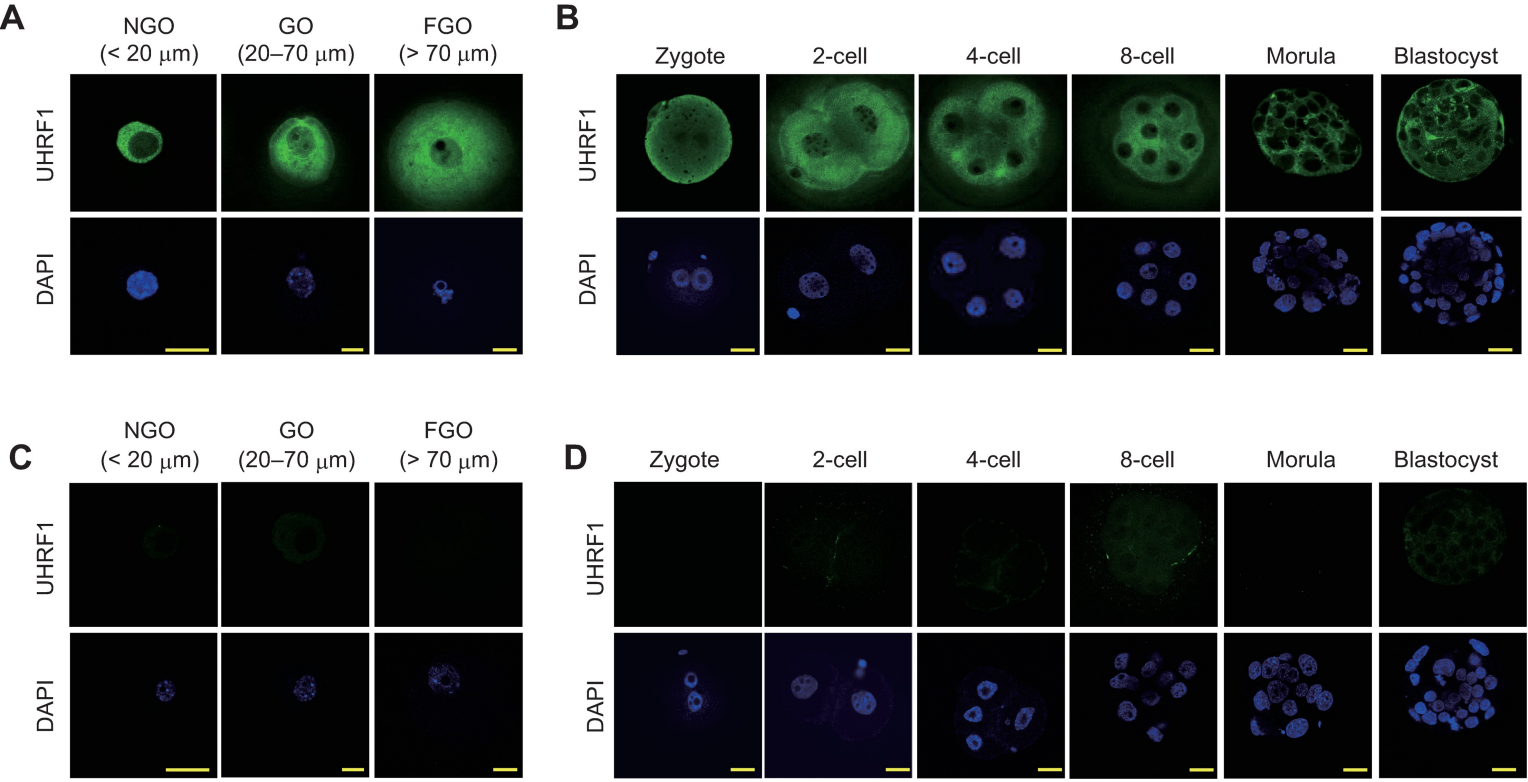
Expression and subcellular localization of UHRF1 in oocytes and preimplantation embryos. (A,B) Immunostaining of C57BL/6J developing oocytes (A), zygote to blastocyst stage embryos (B), *Uhrf1* KO oocytes (C), and *Uhrf1* mat-KO embryos (D) performed with an anti-UHRF1 antibody (Th-10a [green]). Each experiment was done with at least two batches of oocytes/embryos, and the total number of oocytes/embryos examined at each stage exceeded 20. The cell nucleus was counterstained with DAPI (blue). NGO, <20 μm; GO, 20–50 μm; FGO, >70 μm. Scale bar, 20 μm.

To reveal the role of UHRF1 proteins in mouse oocytes and preimplantation embryos, we first examined the expression and subcellular localization of UHRF1 by immunostaining. Like DNMT1 [31], UHRF1 was mainly localized in the cytoplasm of GOs and preimplantation embryos (Fig 1A and 1B). However, a weaker signal was detected in the nucleus. To examine the timing of the zygotic activation of the *Uhrf1* and *Dnmt1* genes, we reprocessed published polyA(+) mRNA-seq data from F1 hybrid embryos [46], where expressed alleles could be distinguished based on the presence of single nucleotide polymorphisms (SNPs). While the levels of maternal *Uhrf1* and *Dnmt1* mRNAs had decreased by the 16-cell stage, the total levels of both mRNAs increased after this stage (S1 Fig). The *Uhrf1* and *Dnmt1* mRNAs, which were derived from the paternal allele, were first detected at the mid 2-cell stage (S1 Fig), pinpointing the timing of the zygotic activation of these genes.

We generated oocyte-specific *Uhrf1* KO mice carrying a *zona pellucida glycoprotein 3* (*Zp3*)-Cre transgene [*Uhrf1*^2lox/2lox^, *Zp3*-Cre] (S2A and S2B Fig). The *Zp3*-Cre transgene is expressed exclusively in GOs [47]. No significant defects were observed in the morphology, size, or number of *Uhrf1* KO [*Uhrf1*^1lox/1lox^] FGOs (S2C Fig). As expected, virtually no *Uhrf1* mRNA (by a quantitative reverse transcription-polymerase chain reaction; qRT-PCR) or UHRF1 protein (by Western blotting) was detected in the KO FGOs (S2D and S2E Fig). The predicted truncated protein (amino acid 1–133) was not expressed, as its coding exons (2–3) were not detected at the mRNA level (S2D Fig), probably due to nonsense-mediated mRNA decay [48]. The loss of UHRF1 protein was confirmed by immunostaining as early as the non-growing oocyte (NGO) stage (Fig 1C and S2F Fig). The expression of *Dnmt1* was not affected by the *Uhrf1* KO (S2E Fig). When *Uhrf1* KO oocytes were fertilized with wild-type sperm *in vitro* (*in vitro* fertilization, IVF), only 20% of the embryos (*Uhrf1* maternal-KO or mat-KO [*Uhrf1*^1lox/+^]) developed to the expanded blastocyst stage (S3A Fig), suggesting that maternal UHRF1 plays a critical role in preimplantation development. This phenotype was much more severe than that of *Dnmt1* mat-KO [*Dnmt1*^1lox/+^] embryos [31,32] (S3A Fig). Virtually no UHRF1 protein was detected by immunostaining even in the surviving embryos (Fig 1D).

**Fig 2:**
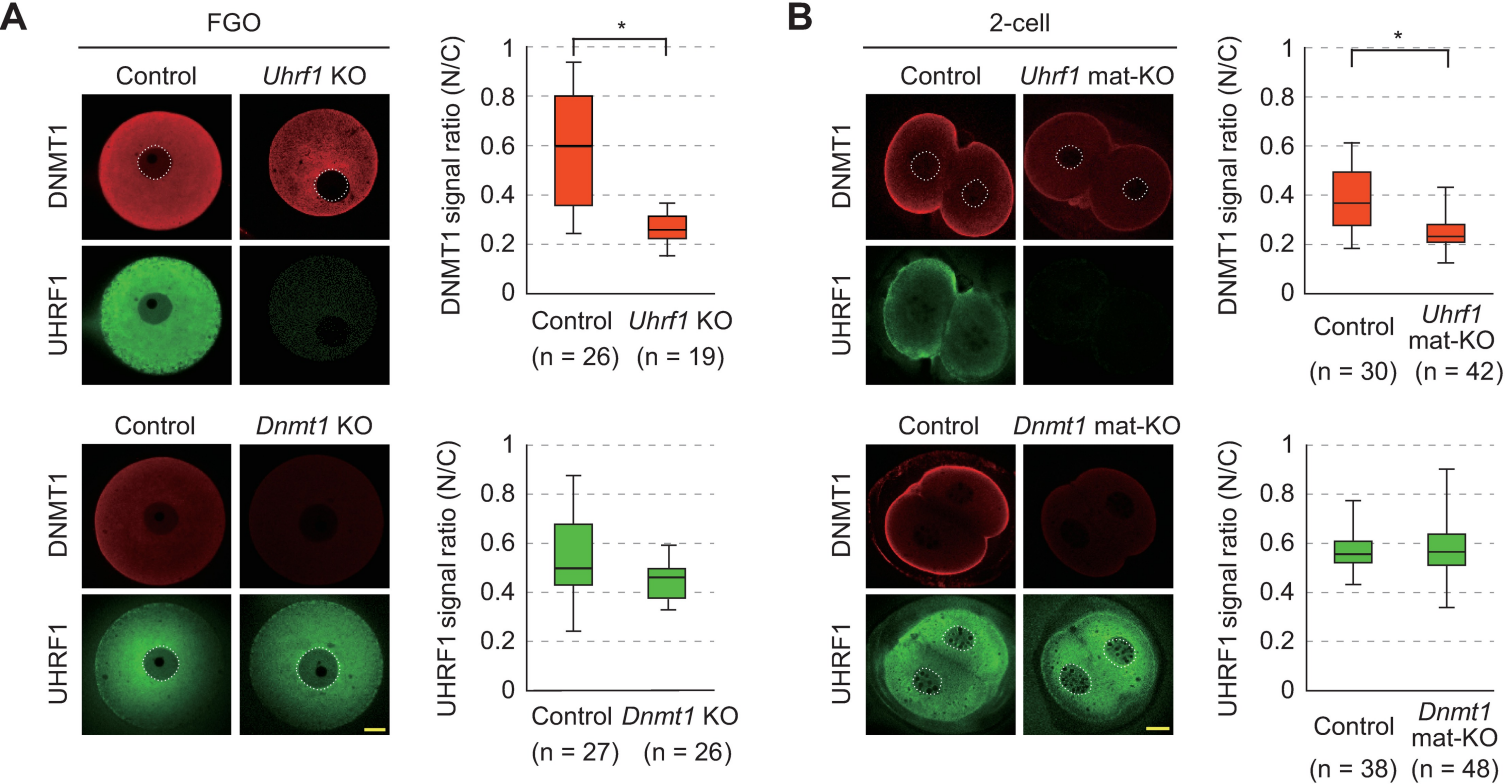
Dependence of nuclear localization of DNMT1 on UHRF1. (A,B) Immunostaining of control [*Uhrf1*^2lox/2lox^ or *Dnmt1*^2lox/2lox^], *Uhrf1* KO, and *Dnmt1* KO FGOs (A) and control [*Uhrf1*^2lox/+^ or *Dnmt1*^2lox/+^], *Uhrf1* mat-KO, and *Dnmt1* mat-KO 2-cell embryos (B) was performed with anti-UHRF1 antibodies (green) or anti-DNMT1 antibodies (red). The nuclear/cytoplasmic signal ratios (N/C) were calculated using the mean signal intensities obtained by linear scanning across the cells. Each experiment was done with at least two batches of oocytes/embryos, and the total number (n) of oocytes/embryos is indicated. Each box indicates the 25–75 percentile and the bar in each box indicates the median. Asterisk, *P* <0.01 (Mann-Whitney *U* test).

Interestingly, while the absence of DNMT1 did not affect the subcellular localization of UHRF1, the absence of UHRF1 clearly decreased the level of DNMT1 in the nucleus of FGOs (Fig 2A). The absence of maternal UHRF1 also decreased the level of nuclear DNMT1 in 2-cell embryos (Fig 2B), suggesting that the nuclear localization of DNMT1 is dependent on UHRF1.

### The role of maternal UHRF1 in the CG maintenance methylation in preimplantation embryos

In preimplantation embryos, both the paternal and maternal genomes are globally demethylated, while the ICRs and certain retrotransposons (such as intracisternal A particle [IAP] elements) retain CG methylation [4,23,31,49,50]. To examine whether the maternal UHRF1 proteins play a role in CG maintenance methylation in these sequences, whole genome bisulfite sequencing (WGBS) was performed with control [*Uhrf1*^2lox/+^], *Uhrf1* mat-KO, and *Dnmt1* mat-KO blastocysts generated by IVF. Only well-developed *Uhrf1* mat-KO blastocysts were pooled (S3A Fig, indicated as expanded) and subjected to the analysis. Since KO females were of the C57BL/6J background (*Mus musculus domesticus*) and the wild-type sperm was from JF1 males (*Mus musculus molossinus*), the parental alleles were distinguishable where SNPs were available. We used the post-bisulfite adaptor tagging (PBAT) method, which was amplification-free and applicable to a limited amount of DNA, to construct the WGBS libraries [51].

**Fig 3:**
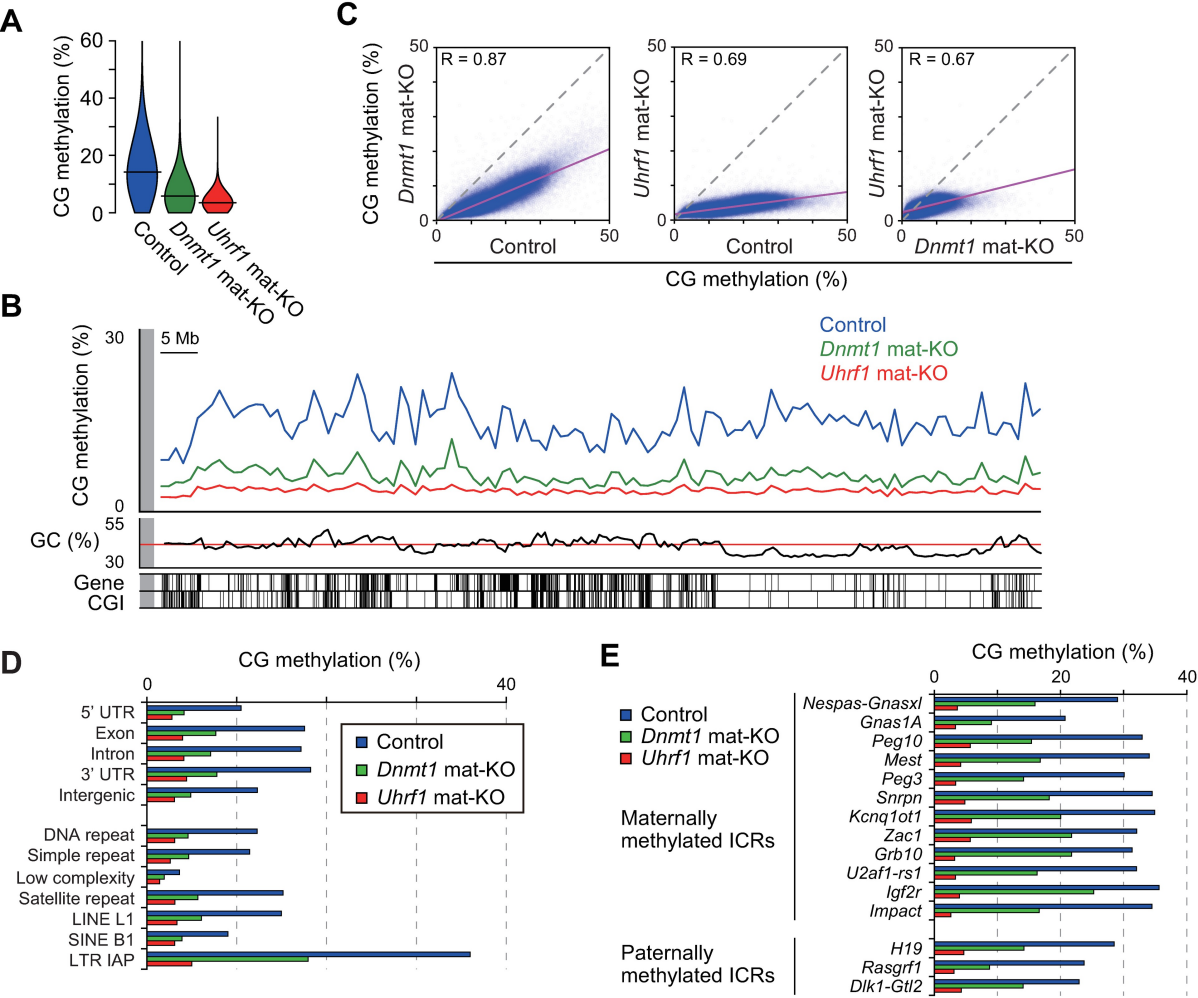
The role of maternal UHRF1 in CG maintenance methylation in preimplantation embryos. (A) Violin plots showing the distribution of the 10-kb genomic regions with different CG methylation levels in control [*Uhrf1*^2lox/+^], *Uhrf1* mat-KO, and *Dnmt1* mat-KO blastocysts. The horizontal bars indicate the mean CG methylation levels. (B) The genomic distribution of CG methylation across chromosome 14 in control and mat-KO blastocysts. The CG methylation levels of 1-Mb windows are shown. GC contents, RefSeq genes, and CGIs are shown at the bottom. (C) The correlations between the CG methylation levels of the 10-kb genomic windows in the control and mat-KO blastocysts. (D) The CG methylation levels of respective portions of the genic regions, intergenic regions, and various repetitive sequences in control and mat-KO blastocysts. (E) The CG methylation levels of the ICRs in control and mat-KO blastocysts.

Our WGBS with replicate samples basically confirmed good reproducibility, but the *Uhrf1* mat-KO blastocysts showed a lower correlation, likely due to slight differences in developmental stage and extremely low levels of CG methylation, which are sensitive to small variations (S1 and S2 Tables and S4 Fig). The global CG methylation levels of the control, *Uhrf1* mat-KO, and *Dnmt1* mat-KO blastocysts were 14.3%, 3.5%, and 5.7%, respectively (Fig 3A and S2 Table, combined replicate data). Thus, the absence of maternal UHRF1 reduced the level of global CG methylation to one-fourth of the control level. The fact that the global methylation decreased by more than 50% suggests that not only the maternal genome but also the paternal genome, which was derived from the wild-type sperm, was affected after fertilization. Indeed, this was confirmed by the allele-specific methylation analysis using SNPs (see later). The loss of CG methylation was milder in *Dnmt1* mat-KO blastocysts. The non-CG methylation level was basically unaffected by the absence of either maternal UHRF1 or maternal DNMT1 (S2 Table).

The analysis of CG methylation in 1-megabase (Mb) or 10-kilobase (kb) windows revealed that the absence of maternal UHRF1 or DNMT1 caused a basically global and proportional decrease in CG methylation across the genome (Fig 3B and 3C and S3B Fig). The genic regions, intergenic regions, repetitive elements (including IAPs), and CpG islands (CGIs) were all affected (Fig 3D and S3C Fig). The expected allele-specific CG methylation was confirmed in 12 (ten maternally methylated and two paternally methylated) of the 15 ICRs examined in the control blastocysts (S3D Fig); SNPs were not available for the rest of the ICRs. As we reported previously [31], the level of CG methylation was decreased to half that of the control blastocysts at the ICRs in *Dnmt1* mat-KO blastocysts. In contrast, the CG maintenance methylation at the ICRs was more severely affected in *Uhrf1* mat-KO blastocysts, to less than one-fourth the level observed in the control blastocysts (Fig 3E and S3D Fig). The loss of methylation from both parental genomes in mat-KO blastocysts indicates a role for maternal Uhrf1 in the maintenance of CG methylation.

### The role of UHRF1 in *de novo* CG methylation during oocyte growth

**Fig 4:**
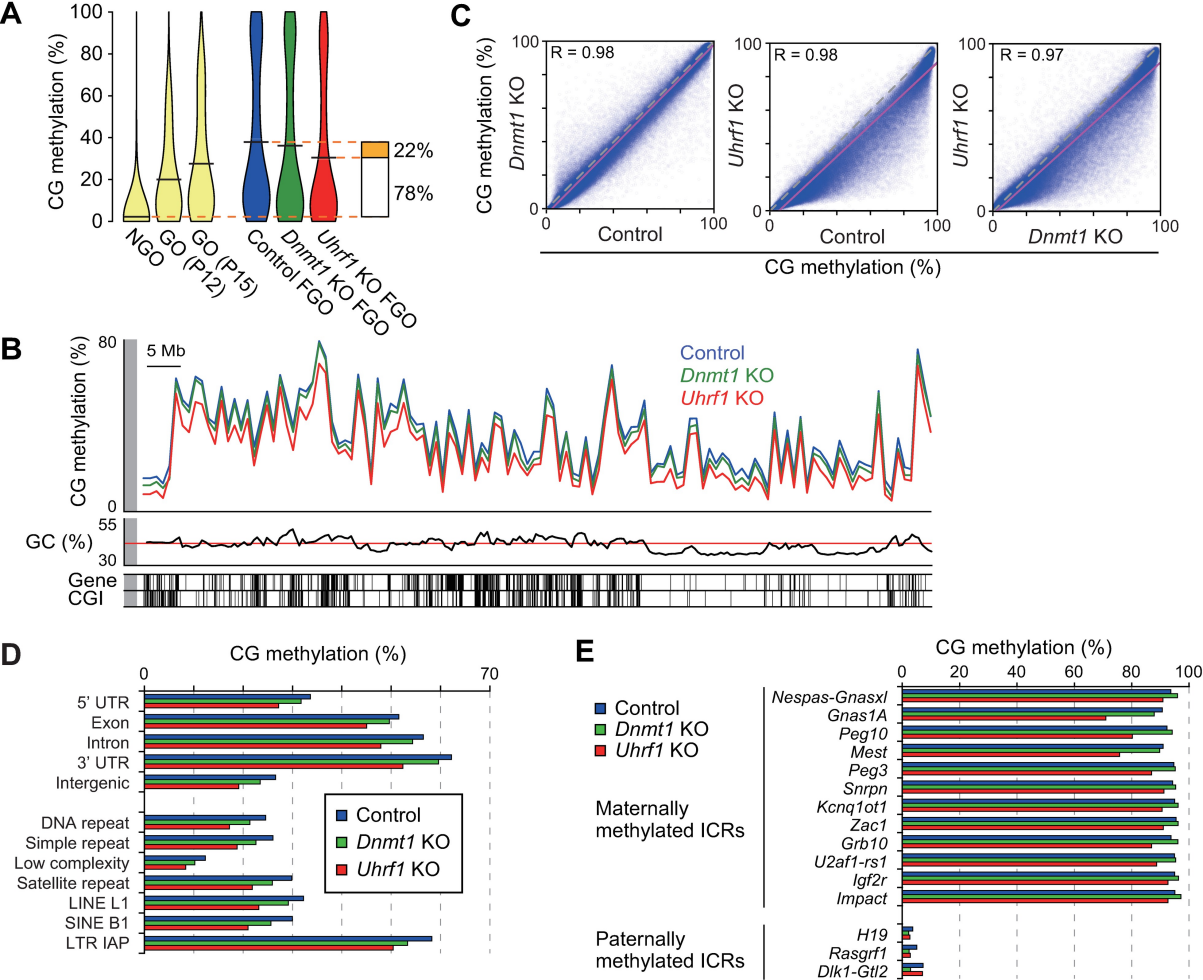
The role of UHRF1 in *de novo* CG methylation during oocyte growth. (A) Violin plots showing the distribution of the 10-kb genomic regions with different CG methylation levels in NGOs [5], GOs (P12 and P15) [53], and control [*Uhrf1*^2lox/2lox^], *Dnmt1* KO, and *Uhrf1* KO FGOs. The horizontal bars indicate the mean CG methylation levels. (B) The genomic distribution of CG methylation across chromosome 14 in control and KO FGOs. The CG methylation levels of the 1-Mb windows are shown. GC contents, RefSeq genes, and CGIs are shown at the bottom. (C) The correlations between the CG methylation levels of the 10-kb genomic windows in control and KO FGOs. (D) The CG methylation levels of respective portions of the genic regions, intergenic regions, and various repetitive sequences in control and respective KO FGOs. (E) The CG methylation levels of the ICRs in control and KO FGOs.

We next examined whether this protein has any effect on CG methylation in FGOs. We therefore performed WGBS with control [*Uhrf1*^2lox/2lox^] and *Uhrf1* KO FGOs (S1 and S2 Tables and S4 Fig). We found that the global CG methylation level was 30.8% in *Uhrf1* KO FGOs, which was 7.9% lower in comparison to control FGOs (38.7%) (Fig 4A and S2 Table). This was surprising as no DNA replication occurs during oocyte growth (meiotic prophase I), during which only *de novo* methylation is observed. Since the CG methylation level in NGOs was reported to be 2.3% [5], these results suggested that UHRF1 was required for approximately one-fourth of the total increase in CG methylation (Fig 4A). We previously reported that the slight decrease in CG methylation in *Dnmt1* KO FGOs (36.0% global CG methylation level compared to 38.7% in the control FGOs) (Fig 4A and S2 Table) could be explained by increased hemimethylation [5]. Although the proportion of hemimethylated sites among the highly methylated CG sites was 6.1% greater in *Uhrf1* KO FGOs (12.9%) than it was in *Dnmt1* KO FGOs (6.8%) (S5A Fig) (see Materials and Methods), this difference was not sufficient to explain the decrease in CG methylation that was observed in the *Uhrf1* KO FGOs.

We previously reported that, in FGOs, nearly 65% of all 5mCs occurred at non-CG sites [5]. This was confirmed in our control FGOs (S5B Fig and S2 Table) and, surprisingly, non-CG methylation was decreased to 85% of the level of control FGOs in *Uhrf1* KO FGOs (S5B–S5D Fig and S2 Table). This corresponded to a 0.6% reduction in non-CG methylation on a per site basis (from 3.2% to 2.6%) (S2 Table). *Dnmt1* KO FGOs showed an increased non-CG methylation level, possibly due to the compensatory upregulation of *Dnmt3a* [5]. With regard to the sequence context, the CHG and CHH sites (where H represents the base A, C, or T) were equally affected (S5B Fig). These results strongly suggest that UHRF1 plays a role in *de novo* CG and non-CG methylation, not only through the recognition of hemimethylated CG sites, but also through an unknown mechanism.

To examine whether any regions of the genome are preferred for the *de novo* CG methylation involving UHRF1, we determined the CG methylation levels in 1-Mb windows and found that the levels in *Uhrf1* KO FGOs decreased across the genome in comparison to control FGOs (Fig 4B and S6A Fig). However, subsequent analyses in 10-kb windows revealed that the loss of CG methylation was most remarkable in genomic regions that showed moderate levels of CG methylation (Fig 4C). In addition, there was a positive correlation between the losses in CG and non-CG methylation in the 10-kb genomic windows (S6B Fig). Various repetitive elements, including IAPs, were moderately affected (Fig 4D). Many CGIs were known to be methylated in FGOs [49] and, again, moderately methylated CGIs were the most affected in *Uhrf1* KO FGOs (S6C Fig). In contrast, the maternally methylated ICRs, which represent the most highly methylated regions in the FGO genome, were only slightly affected by the absence of UHRF1, except the *Gnas1A*, *Peg10*, and *Mest* ICRs, which showed significant reduction in CG methylation (p = 6.49 x 10^−121^, 1.85 x 10^−41^, and 2.68 x 10^−81^, respectively; kai-squared test) (Fig 4E).

### UHRF1 facilitates *de novo* methylation in inactive regions during late oocyte growth

**Fig 5:**
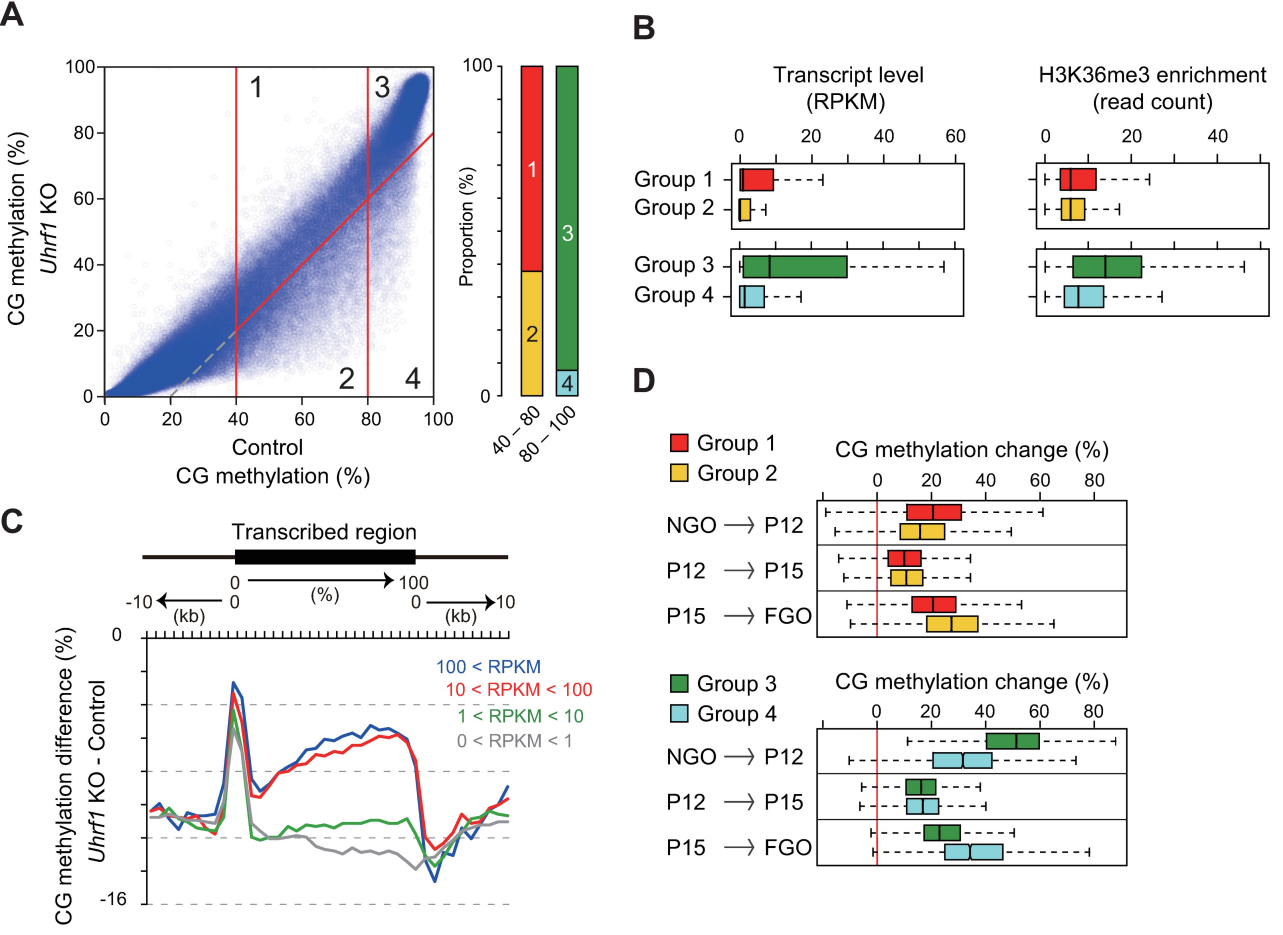
UHRF1 facilitates *de novo* methylation in inactive regions during late oocyte growth. (A) The classification of the 10-kb genomic regions that were moderately (40–80%) or highly (≥80%) methylated in the control FGOs, based on the extent of methylation change (≥20% or not) in *Uhrf1* KO FGOs (Groups 1–4). The right panel shows the proportion of moderately and highly methylated 10-kb regions belonging to each group. (B) The distribution of the transcript levels (RPKM) and H3K36me3 enrichment levels [20] (corrected read count) of the 10-kb regions belonging to each group. The bar in each box indicates the median value. (C) Differences in CG methylation across the transcribed regions between control and *Uhrf1* KO FGOs. The transcribed regions were classified into four categories based on the expression levels (RPKM) in control FGOs and the results for the respective groups are shown separately. (D) The changes in CG methylation occurring in the three different stages of oocyte growth were determined for the 10-kb regions of the respective groups.

As a first step to understand how UHRF1 is involved in *de novo* CG and non-CG methylation in oocytes, we attempted to explore the features of the regions affected in *Uhrf1* KO FGOs in greater detail. We first identified the genomic regions (10-kb windows) with a CG methylation level of ≥40% in control FGOs and divided them into four groups: moderately (40–80%) methylated regions that were unaffected (decrease <20%) by *Uhrf1* KO (Group 1); moderately methylated regions that were affected (decrease ≥20%) by KO (Group 2); highly (≥80%) methylated regions that were unaffected by KO (Group 3); highly methylated regions that were affected by KO (Group 4) (Fig 5A and S3 Table). Of the moderately methylated regions (Groups 1 and 2), 35% of the cases were affected (Group 2) but, in contrast, only 9% of the highly methylated regions (Groups 3 and 4) were affected (Group 4) (Fig 5A), confirming that the moderately methylated regions were preferentially affected.

Transcription-coupled *de novo* CG methylation is predominant in oocytes [4,18,19]. To examine the relationship between transcription and UHRF1-dependent *de novo* CG methylation, we performed RNA-seq with replicate samples from control [*Uhrf1*^2lox/2lox^] and *Uhrf1* KO FGOs (S4 Table). Overall, the transcription profiles of these FGOs were very similar (R = 0.969, combined replicate data, S6D Fig), and we confirmed that the highly methylated regions produced more transcripts (Fig 5B). Also, using previously published H3K36me3 chromatin immunoprecipitation-sequencing data from GOs (≥30–65 μm) [20], we found that the highly methylated regions were preferentially marked with this transcription-coupled histone mark [52] (Fig 5B). We then examined the CG methylation changes in *Uhrf1* KO FGOs in the transcribed regions and found that untranscribed or only lowly transcribed regions were the most severely affected (Fig 5C).

Lastly, we used the published WGBS data from NGOs collected at the newborn stage [5] and GOs collected on postnatal days 12 (P12) and P15 [53] to examine the timing of *de novo* CG methylation in affected and unaffected regions. In comparison to the regions that were unaffected by *Uhrf1* KO (Groups 1 and 3), the affected regions gained less methylation in the early phase of oocyte growth (between newborn NGO and P12 GO) and underwent greater methylation in the later phase (between P15 GO to FGO) (Fig 5D). Interestingly, the three maternally methylated ICRs more affected by *Uhrf1* KO (the *Gnas1A*, *Peg10*, and *Mest* ICRs) compared to others (Fig 4E) also gained more methylation in the later phase (S6E Fig). These results indicate that UHRF1 accelerates *de novo* CG methylation in untranscribed or lowly transcribed regions during the late stage of oocyte growth.

### The relative contribution to *de novo* CG methylation and maintenance CG methylation

Since UHRF1 was found to be involved in *de novo* CG (and non-CG) methylation in GOs, we attempted to determine what proportion of the loss of CG methylation in *Uhrf1* mat-KO blastocysts (Fig 3) was explained by the *de novo* activity and what proportion was explained by the maintenance activity. The *de novo* activity only affects the maternal genome, while the maintenance activity affects both parental genomes: we therefore determined the CG methylation level of each parental genome in control and *Uhrf1* mat-KO blastocysts using SNPs. In the control blastocysts, the CG methylation level of the maternal genome was 14.3%, which was 2.3% lower than that in the paternal genome (16.6%) (S7A Fig). (The observed levels were lower than those of the inner cell mass cells (20% and 21% for the maternal and paternal genome, respectively) [50], perhaps because trophectoderm cells display lower methylation.) In *Uhrf1* mat-KO blastocysts, the CG methylation levels of the maternal and paternal genomes were decreased to 3.1% and 4.5%, respectively (S7A Fig). (Since SNP-associated CG sites were more frequently found in the genic regions, where the CG methylation level was high, the average CG methylation level detected in the parental genomes (3.8%) was slightly higher than that in the whole genome (3.5%).)

The CG methylation level in control and *Uhrf1* KO FGOs was 38.7% and 30.8%, respectively (S2 Table). These levels correspond to those of the maternal genome at the time of fertilization. Since the methylation level of the maternal genome was 14.3% in control blastocysts (see above), this genome undergoes a ×0.37 (14.3%/38.7%) methylation change, mainly due to replication-coupled dilution during preimplantation development (S7B Fig). (Hydroxylation-dependent demethylation selectively occurs on the paternal genome [24–27].) If there was no maternal effect caused by oocyte-specific *Uhrf1* KO and if we apply the demethylation rate determined above to the maternal genome derived from *Uhrf1* KO FGOs, then the expected methylation level would be 11.4% (= 30.8% ×0.37) (S7B Fig). Thus, the *de novo* activity would account for 2.9% (= 14.3%–11.4%) of the total methylation loss of the maternal genome (11.2% [= 14.3%–3.1%]) in *Uhrf1* mat-KO blastocysts). The rest of the loss (8.3% [= 11.2%–2.9%]) would be attributable to the maintenance activity in preimplantation embryos (S7B Fig). Although the precise values may be subject to variations between the samples (S4 Fig), these results show that the loss of CG methylation in the *Uhrf1* mat-KO blastocysts can largely be explained by the loss of the maintenance activity in early embryos.

## Discussion

In the present study, we found that the UHRF1 produced in oocytes has an important role in shaping the DNA methylation landscape in oocytes and preimplantation embryos. We first showed that, like DNMT1 [31], UHRF1 is mainly, but not exclusively, localized in the cytoplasm during oocyte growth and preimplantation development. Why should epigenetic regulators, which in most cases exert their function in the nucleus, exist in the cytoplasm during this period? For DNMT1, a few studies have addressed the mechanism of cytoplasmic localization in early embryos [54]; however its function in the cytoplasm is still unknown. Furthermore, CG maintenance methylation and other events that are known to involve UHRF1 (for example, DNA repair [55–57]) occur in the nucleus and thus the biological significance of cytoplasmic UHRF1 remains to be clarified. As expected, however, a small amount of UHRF1 did exist in the nucleus of oocytes and early embryos, as did DNMT1 [31]. Interestingly, the nuclear localization of DNMT1 was dependent on UHRF1, as oocyte-specific *Uhrf1* KO resulted in a greatly reduced DNMT1 signal in the nucleus. A previous study in ESCs showed that DNMT1 recruitment to specific target loci, but not nuclear localization, is dependent on UHRF1 [10,11]. Thus, the nuclear entry and/or retention of DNMT1 is regulated differently in different cell types.

*Uhrf1* mat-KO embryos showed partial preimplantation lethality, a phenotype that is much more severe than that of *Dnmt1* mat-KO embryos. Our WGBS showed that the global CG methylation level is more greatly reduced in *Uhrf1* mat-KO blastocysts (×0.25 in comparison to control blastocysts) than it is in *Dnmt1* mat-KO blastocysts (×0.36 in comparison to control). The loss of CG methylation at the ICRs and IAP elements in the *Dnmt1* mat-KO embryos was approximately ×0.50 (see also [31]); however, the loss in *Uhrf1* mat-KO embryos was greater (×0.25 or less). These results strongly suggest that maternal UHRF1 is essential for imprint maintenance and IAP repression in preimplantation embryos. It is known that the repressed alleles of the ICRs and IAP elements are marked with H3K9me3 in ESCs and preimplantation embryos [58,59]. These results suggest that UHRF1 may be recruited to specific targets through H3K9me3 recognition via its TTD [35,36,42,43]. However, mice expressing a mutated UHRF1 protein with a defective TTD exhibit normal viability/fertility with an only ~10% reduction in CG methylation [60]. Thus, the mechanism through which UHRF1 is recruited to the targets (particularly the ICRs and IAP elements) in the preimplantation embryos remains an open question.

An unexpected discovery of the present study was the involvement of UHRF1 in *de novo* CG and non-CG methylation in GOs. We previously reported that DNMT1 is important for the completion of *de novo* CG methylation in oocytes: DNMT1 appears to act on the hemimethylated CG sites left behind during the *de novo* methylation process and make them fully methylated [5]. UHRF1 may contribute to this process by efficiently recognizing hemimethylated CG sites since the proportion of hemimethylated CG sites among the highly methylated sites was increased in *Uhrf1* KO FGOs. This increase in hemimethylated CG sites was greater than that observed in *Dnmt1* KO FGOs. Unexpectedly, but consistent with the above-mentioned findings, we found that not only CG methylation, but also non-CG methylation, was affected in *Uhrf1* KO FGOs. Thus we speculate that UHRF1 may be directly involved in *de novo* CG and non-CG methylation, which are both mediated by the DNMT3A-DNMT3L complex in GOs [5].

Since UHRF1 can interact with DNMT3A and DNMT3B in ESCs and silence exogenous promoters [61], it may interact with these enzymes in oocytes as well and facilitate *de novo* methylation. Interestingly, however, UHRF1 only played a limited role in the establishment of CG methylation at the maternally methylated ICRs: rather, the protein was important for *de novo* CG and non-CG methylation in the moderately methylated genomic regions. These regions were mostly untranscribed, lacked H3K36me3 marking, and tended to be *de novo* methylated in a late stage of oocyte growth. Interestingly, among the maternally methylated ICRs, those that were less resistant to UHRF1 depletion normally gained CG methylation in the late stage of oocyte growth. At present, the mechanism through which UHRF1 is recruited to these regions and through which it engages in *de novo* CG and non-CG methylation remains to be elucidated.

In conclusion, our study showed the importance of maternal UHRF1 in CG maintenance methylation during preimplantation development and in *de novo* CG and non-CG methylation in transcriptionally inactive regions during oocyte growth. To the best of our knowledge, this is the first study to show the involvement of UHRF1 in *de novo* methylation *in vivo*. We believe that our findings give further insight into the epigenetic reprogramming in early development and provide a basis for further improvements in reproductive technologies and regenerative medicine.

## Materials and methods

### Ethics statement

Mouse husbandry and all of the mouse experiments were carried out under the ethical guidelines of Kyushu University. The mice were euthanized by carbon dioxide asphyxiation. The protocols were approved by the Institutional Animal Care and Use Committee of Kyushu University.

### KO mice

We generated ESCs carrying a loxP site in intron 3 and a neomycin selection cassette flanked by loxP sites in intron 5 of *Uhrf1* [*Uhrf1*^3lox/+^]. *Uhrf1*^3lox/+^ mice were obtained by chimera formation and germline transmission. Because the *Uhrf1*^3lox/3lox^ males were infertile, we crossed females of this genotype with EIIA-Cre males [62] to remove the neomycin cassette and generated *Uhrf1*^2lox/+^ mice. *Uhrf1*^2lox/2lox^ mice were crossed with mice carrying the *Zp3*-Cre transgene [47] to generate oocyte-specific *Uhrf1* KO mice [*Uhrf1*^2lox/2lox^, *Zp3*-Cre]. The oocyte-specific *Dnmt1* KO mice [*Dnmt1*^2lox/2lox^, *Zp3*-Cre] have been described previously [31,63]. The mice were genotyped by PCR under standard conditions using the primers listed in S5 Table. All of the KO mice were basically of the C57BL/6J background (*Mus musculus domesticus*).

### Oocyte collection, IVF, and embryo culture

NGOs and GOs were obtained from the ovaries on P10. FGOs were obtained from the ovaries at 8–12 weeks. Preimplantation embryos were obtained by IVF. Females aged ≥8 weeks were injected with 7.5 U of pregnant mare serum gonadotropin; 48 h later, they were injected with 7.5 U of human chorionic gonadotropin to induce super ovulation. Cumulus-oocyte complexes were collected from the oviduct and fertilized with C57BL/6J sperm for immunostaining and Western blotting or with JF1 (*Mus musculus molossinus*) sperm for WGBS. Cumulus cells were carefully removed by washing with phosphate-buffered saline (PBS), and fertilized embryos were cultured in KSOM medium (EmbryoMax KSOM Medium (1X) w/ 1/2 Amino Acids, Merck Millipore) at 37°C with 5% CO_2_.

### Western blotting

Twenty FGOs were lysed in a sample buffer (62.5 mM Tris-HCl (pH 6.8), 0.5× PBS, 2% SDS, 10% glycerol, and 5% 2-mercaptoethanol) and sonicated. Proteins were denatured by heating at 95°C for 5 min, separated by electrophoresis on a 10% SDS polyacrylamide gel, and transferred onto a nitrocellulose membrane (Amersham). The blots were blocked with 5% skimmed milk, incubated with anti-UHRF1 rabbit polyclonal antibody (M-132, Santa Cruz), anti-DNMT1 rabbit polyclonal antibody (a kind gift from Shoji Tajima), or anti-β-actin mouse monoclonal antibody (AC-15, Santa Cruz) (1:10,000 dilution each). After several washes, the blots were incubated with HRP-conjugated anti-rabbit or mouse IgG antibody (1:10,000 dilution), and detected using Chemi-Lumi One Ultra reagent (11644–40, Nacalai Tesque) and an LAS-3000 lumino-image analyzer (Fujifilm).

### Immunostaining

Embryos and oocytes were fixed with 4% paraformaldehyde in PBS at room temperature for 30 min and washed with PBS three times. After incubation with a pretreatment buffer (1% bovine serum albumin and 2% Triton-X100 in PBS) at room temperature for 15 min, the embryos and oocytes were incubated at 4°C overnight with anti-UHRF1 rat monoclonal (Th-10a, MBL) antibody (1:500 dilution) or anti-DNMT1 rabbit polyclonal antibody (1:1000 dilution). After washing three times, the embryos and oocytes were incubated with fluorescence-labeled secondary antibody (1:1000 dilution) at room temperature for 30 min. The secondary antibody was CF488 donkey anti-rabbit IgG (H+L) antibody (20015, Biotium Inc), CF488A donkey anti-rat IgG (H+L) antibody (20027, Biotium Inc), or CF594 donkey anti-rabbit IgG (H+L) antibody (20152, Biotium Inc). After washing three times, oocytes and embryos were mounted in VECTASHIELD medium with DAPI (Vector Laboratory) and observed using an LSM510 or LSM700 confocal laser scanning microscope (Carl Zeiss) with a 63× objective lens and a 10× ocular lens. The signal intensities were measured using the ZEN 2012 (blue edition) software program (Carl Zeiss). The nuclear/cytoplasmic signal ratios were calculated using the mean nuclear and cytoplasmic signal intensities.

### The qRT-PCR

Total RNA was extracted from 100 FGOs using AllPrep DNA/RNA Mini kit (QIAGEN) and reverse transcribed using a PrimeScript RT reagent kit with gDNA Eraser (TaKaRa). A qRT-PCR was carried out using a KAPA SYBR Fast qPCR kit (Kapa Biosystems) in Thermal Cycler Dice Real Time System Single (TaKaRa) in accordance with the manufacturer's protocol. The primers that were used are listed in S5 Table. The relative gene expression was quantified using the comparative cycle threshold method, with the *Gapdh* expression used for normalization.

### WGBS and the data analysis

WGBS libraries were prepared using the PBAT method as described in our previous reports [5,51,64]. Five hundred to 1,000 oocytes or 30–40 blastocysts were spiked with 0.1 ng of lambda phage DNA (Promega) and subjected to bisulfite conversion. The concentrations of the PBAT libraries were measured by a qPCR using a KAPA Illumina Library Quantification kit (Kapa Biosystems). Cluster generation and sequencing were performed in a single-read mode using a TruSeq SR Cluster kit v3-cBot-HS (Illumina) and TruSeq SBS kit v3-HS (Illumina) according to manufacturer’s protocols. The libraries were sequenced on a HiSeq 2500 or HiSeq 1500 equipped with HCS v2.0.5 and RTA v1.17.20 (suitable for WGBS) [65] to generate 101-nucleotide single-end reads. We trimmed the raw sequence reads to 96 bases by removing the adapter sequences from the 5’ end and one base from the 3’ end. The resulting reads were aligned to the reference mouse genome (mm10) using Bismark v0.14.2 [66]. The seed length was 28, the maximum number of mismatches permitted in the seed was 1, and the “—pbat” option was selected. Only uniquely aligned reads were analyzed. We estimated the bisulfite conversion rate using reads that were uniquely aligned to the lambda phage genome (accession no. J02459). The sequences and information of the chromosomes, RefSeq genes, CGIs, and repetitive elements were downloaded from the UCSC genome browser [67]. Throughout this paper, the global CG methylation levels refer to the weighted levels, which take the sequencing depth into account [68].

### Estimating the frequency of hemimethylated CG sites

The proportion of hemimethylated sites among the highly methylated CG sites was estimated as previously described [5]. In brief, we selected CG sites with a methylation level of ≥70% on either strand that showed a read depth of ≥10. The CG sites with a significant difference in methylation (Fisher’s exact test, p <0.05) were defined hemimethylated.

### RNA-seq and the data analysis

Total RNA was extracted from 30 oocytes. Whole transcript amplification and library construction were performed as described previously [69]. The libraries were sequenced on a HiSeq 2000 to generate 50-nucleotide single-end reads. We trimmed the raw sequence reads by removing poly-A tails and low quality bases from the 3’ end. The resulting sequences were aligned to the reference mouse genome (mm10) using the TopHat software program (v2.0.14) [70]. We used a gtf file of published oocyte transcripts [19] for mapping. The mapped reads were counted using featureCounts [71] to calculate the reads per kb per million mapped reads (RPKM) values.

### Accession numbers

The sequence data sets supporting the results of this article are available in the DDBJ Sequence Read Archive under accession number DRA005849.

## Supporting information

S1 Fig The expression of *Uhrf1* and *Dnmt1* mRNAs in preimplantation embryos. The expression of *Uhrf1* and *Dnmt1* mRNAs from the maternal and paternal alleles was analyzed separately using published data from (CAST/EiJ × C57BL/6J) F1 hybrid embryos (GSE45719) [46]. An enlarged view of the *Uhrf1* and *Dnmt1* expression from the zygote to the late 4-cell stage is shown on the right. The expression levels are shown by the RPKM values. Error bar, standard error.
(EPS)

S2 Fig The establishment of oocyte-specific *Uhrf1* KO mice. (A) A schematic representation of the genomic organization of the wild-type (WT), 2lox, and 1lox *Uhrf1* alleles. The filled boxes indicate the protein-coding regions. (B) The predicted domain architecture of the WT and mutated UHRF1 proteins. The immunogen used to generate the anti-UHRF1 antibody M-132 (used for Western blotting) is shown. (C) The morphology of control [*Uhrf1*^2lox/2lox^] and *Uhrf1* KO FGOs. Scale bar, 100 μm. (D) The expression of *Uhrf1* and the *Dnmt* family mRNAs in control and *Uhrf1* KO FGOs was measured by qRT-PCR. Two portions of *Uhrf1* mRNA were amplified for quantification. The expression of *Gapdh* was used for normalization. (E) The detection of UHRF1 and DNMT1o proteins in control and *Uhrf1* KO FGOs by Western blotting using M-132 and anti-DNMT1 antibodies. β-actin was used as a loading control. (F) The immunostaining signal intensities obtained with an anti-UHRF1 antibody (Th-10a) in control and *Uhrf1* KO developing oocytes.
(EPS)

S3 Fig CG methylation in *Uhrf1* mat-KO blastocysts. (A) The morphology of control [*Uhrf1*^2lox/+^], *Dnmt1* mat-KO, and *Uhrf1* mat-KO embryos at 96 h after IVF. Among the *Uhrf1* mat-KO embryos, those that developed to the expanded blastocyst stage are shown on the left. Only well-developed blastocysts were used for WGBS. Scale bar, 100 μm. (B) The genomic distribution of CG methylation across all chromosomes in the control and mat-KO blastocysts. The CG methylation levels of the 1-Mb windows are shown. (C) The correlations between the CG methylation levels of the CGIs in control and mat-KO blastocysts. (D) Allele-specific CG methylation at the ICRs in control and *Uhrf1* mat-KO blastocysts.
(EPS)

S4 Fig Reproducibility of WGBS data between replicates. The correlations of the CG methylation levels of the biological replicates were examined in 10-kb genomic windows.
(EPS)

S5 Fig CG and non-CG methylation in *Uhrf1* KO FGOs. (A) The proportions of hemimethylated sites among highly methylated CG sites in control [*Uhrf1*^2lox/2lox^], *Dnmt1* KO, and *Uhrf1* KO FGOs. (B) The proportion of 5mCs in all Cs in each sequence context. H represents the base A, C, or T. (C) Violin plots showing the distribution of the 10-kb genomic regions with different non-CG methylation levels in control [*Uhrf1*^2lox/+^], *Dnmt1* KO, and *Uhrf1* KO FGOs. The horizontal bars indicate the mean non-CG methylation levels. (D) The correlation between the non-CG methylation levels of the 10-kb genomic windows in the control and KO FGOs.
(EPS)

S6 Fig Genomic regions showing impaired *de novo* CG and non-CG methylation in *Uhrf1* KO FGOs. (A) The genomic distribution of CG methylation across all chromosomes in control [*Uhrf1*^2lox/2lox^] and *Uhrf1* KO FGOs. The CG methylation levels of the 1-Mb windows are shown. (B) Box plots showing the correlation between the impact of *Uhrf1* KO on CG and non-CG methylation in the 10-kb windows. (C) The correlation between the CG methylation levels of CGIs in control and *Uhrf1* KO FGOs. (D) The correlation between the (RPKM+1) values in control and *Uhrf1* KO FGOs. (E) The CG methylation levels of the ICRs in NGOs, P15 GOs, and FGOs.
(EPS)

S7 Fig The contribution of the UHRF1 produced in oocytes to *de novo* CG methylation and maintenance CG methylation. (A) The levels of CG methylation in the respective parental genomes in control [*Uhrf1*^2lox/+^], *Dnmt1* mat-KO and *Uhrf1* mat-KO blastocysts. (B) The contribution of UHRF1 produced in oocytes to *de novo* CG methylation in GOs and maintenance methylation in preimplantation embryos.
(EPS)

S1 Table Sequencing and mapping summary of WGBS. (PDF)

S2 Table Number of methylated cytosines. (PDF)

S3 Table Criteria for Group 1–4 regions. (PDF)

S4 Table Sequencing and mapping summary of RNA-seq. (PDF)

S5 Table List of PCR primers. (PDF)

## References

[1] Smith ZD, Meissner A. DNA methylation: roles in mammalian development. Nat Rev Genet. 2013;14: 204–220. doi: 10.1038/nrg3354 23400093 10.1038/nrg3354

[2] Shen H, Laird PW. Interplay between the cancer genome and epigenome. Cell. 2013;153: 38–55. doi: 10.1016/j.cell.2013.03.008 23540689 10.1016/j.cell.2013.03.008 PMC3648790

[3] Tomizawa S, Kobayashi H, Watanabe T, Andrews S, Hata K, Kelsey G, et al Dynamic stage-specific changes in imprinted differentially methylated regions during early mammalian development and prevalence of non-CpG methylation in oocytes. Development. 2011;138: 811–820. doi: 10.1242/dev.061416 21247965 10.1242/dev.061416 PMC3035086

[4] Kobayashi H, Sakurai T, Imai M, Takahashi N, Fukuda A, Yayoi O, et al Contribution of intragenic DNA methylation in mouse gametic DNA methylomes to establish oocyte-specific heritable marks. PLoS Genet. 2012;8: e1002440 doi: 10.1371/journal.pgen.1002440 22242016 10.1371/journal.pgen.1002440 PMC3252278

[5] Shirane K, Toh H, Kobayashi H, Miura F, Chiba H, Ito T, et al Mouse oocyte methylomes at base resolution reveal genome-wide accumulation of non-CpG methylation and role of DNA methyltransferases. PLoS Genet. 2013;9: e1003439 doi: 10.1371/journal.pgen.1003439 23637617 10.1371/journal.pgen.1003439 PMC3630097

[6] Xie W, Barr CL, Kim A, Yue F, Lee AY, Eubanks J, et al Base-resolution analyses of sequence and parent-of-origin dependent DNA methylation in the mouse genome. Cell. 2012;148: 816–831. doi: 10.1016/j.cell.2011.12.035 22341451 10.1016/j.cell.2011.12.035 PMC3343639

[7] Ramsahoye BH, Biniszkiewicz D, Lyko F, Clark V, Bird AP, Jaenisch R. Non-CpG methylation is prevalent in embryonic stem cells and may be mediated by DNA methyltransferase 3a. Proc Natl Acad Sci U S A. 2000;97: 5237–5242. doi: 10.1073/PNAS.97.10.5237 10805783 10.1073/pnas.97.10.5237 PMC25812

[8] Lister R, Pelizzola M, Dowen RH, Hawkins RD, Hon G, Tonti-Filippini J, et al Human DNA methylomes at base resolution show widespread epigenomic differences. Nature. 2009;462: 315–322. doi: 10.1038/nature08514 19829295 10.1038/nature08514 PMC2857523

[9] Kagiwada S, Kurimoto K, Hirota T, Yamaji M, Saitou M. Replication-coupled passive DNA demethylation for the erasure of genome imprints in mice. EMBO J. 2012;32: 340–353. doi: 10.1038/emboj.2012.331 23241950 10.1038/emboj.2012.331 PMC3567490

[10] Sharif J, Muto M, Takebayashi S, Suetake I, Iwamatsu A, Endo T a, et al The SRA protein Np95 mediates epigenetic inheritance by recruiting Dnmt1 to methylated DNA. Nature. 2007;450: 908–912. doi: 10.1038/nature06397 17994007 10.1038/nature06397

[11] Bostick M, Kim JK, Estève P-O, Clark A, Pradhan S, Jacobsen SE. UHRF1 plays a role in maintaining DNA methylation in mammalian cells. Science. 2007;317: 1760–1764. doi: 10.1126/science.1147939 17673620 10.1126/science.1147939

[12] Yamaguchi S, Shen L, Liu Y, Sendler D, Zhang Y. Role of Tet1 in erasure of genomic imprinting. Nature. 2013;504: 460–464. doi: 10.1038/nature12805 24291790 10.1038/nature12805 PMC3957231

[13] Hackett JA, Sengupta R, Zylicz JJ, Murakami K, Lee C, Down TA, et al Germline DNA demethylation dynamics and imprint erasure through 5-hydroxymethylcytosine. Science. 2013;339: 448–452. doi: 10.1126/science.1229277 23223451 10.1126/science.1229277 PMC3847602

[14] Delaval K, Feil R. Epigenetic regulation of mammalian genomic imprinting. Curr Opin Genet Dev. 2004;14: 188–195. doi: 10.1016/j.gde.2004.01.005 15196466 10.1016/j.gde.2004.01.005

[15] Hata K, Okano M, Lei H, Li E. Dnmt3L cooperates with the Dnmt3 family of de novo DNA methyltransferases to establish maternal imprints in mice. Development. 2002;129: 1983–1993. 11934864 10.1242/dev.129.8.1983

[16] Kaneda M, Hirasawa R, Chiba H, Okano M, Li E, Sasaki H. Genetic evidence for Dnmt3a-dependent imprinting during oocyte growth obtained by conditional knockout with Zp3-Cre and complete exclusion of Dnmt3b by chimera formation. Genes Cells. 2010; 169–179. doi: 10.1111/j.1365-2443.2009.01374.x 20132320 10.1111/j.1365-2443.2009.01374.x

[17] Bourc’his D. Dnmt3L and the Establishment of Maternal Genomic Imprints. Science. 2001;294: 2536–2539. doi: 10.1126/science.1065848 11719692 10.1126/science.1065848

[18] Chotalia M, Smallwood S a, Ruf N, Dawson C, Lucifero D, Frontera M, et al Transcription is required for establishment of germline methylation marks at imprinted genes. Genes Dev. 2009;23: 105–117. doi: 10.1101/gad.495809 19136628 10.1101/gad.495809 PMC2632167

[19] Veselovska L, Smallwood S a., Saadeh H, Stewart KR, Krueger F, Maupetit-Méhouas, et al Deep sequencing and de novo assembly of the mouse oocyte transcriptome define the contribution of transcription to the DNA methylation landscape. Genome Biol. 2015;16: 209 doi: 10.1186/s13059-015-0769-z 26408185 10.1186/s13059-015-0769-z PMC4582738

[20] Stewart KR, Veselovska L, Kim J, Huang J, Saadeh H, Tomizawa S, et al Dynamic changes in histone modifications precede de novo DNA methylation in oocytes. Genes Dev. 2015;29: 2449–2462. doi: 10.1101/gad.271353.115 26584620 10.1101/gad.271353.115 PMC4691949

[21] Dhayalan A, Rajavelu A, Rathert P, Tamas R, Jurkowska RZ, Ragozin S, et al The Dnmt3a PWWP domain reads histone 3 lysine 36 trimethylation and guides DNA methylation. J Biol Chem. 2010;285: 26114–26120. doi: 10.1074/jbc.M109.089433 20547484 10.1074/jbc.M109.089433 PMC2924014

[22] Nashun B, Hill PWS, Smallwood SA, Dharmalingam G, Amouroux R, Clark SJ, et al Continuous histone replacement by Hira is essential for normal transcriptional regulation and de novo DNA methylation during mouse oogenesis. Mol Cell. 2015;60: 611–625. doi: 10.1016/j.molcel.2015.10.010 26549683 10.1016/j.molcel.2015.10.010 PMC4672152

[23] Smith ZD, Chan MM, Mikkelsen TS, Gu H, Gnirke A, Regev A, et al A unique regulatory phase of DNA methylation in the early mammalian embryo. Nature. 2012;484: 339–344. doi: 10.1038/nature10960 22456710 10.1038/nature10960 PMC3331945

[24] Gu T-P, Guo F, Yang H, Wu H-P, Xu G-LG-FG, Liu W, et al The role of Tet3 DNA dioxygenase in epigenetic reprogramming by oocytes. Nature. 2011;477: 606–610. doi: 10.1038/nature10443 21892189 10.1038/nature10443

[25] Wossidlo M, Nakamura T, Lepikhov K, Marques CJ, Zakhartchenko V, Boiani M, et al 5-Hydroxymethylcytosine in the mammalian zygote is linked with epigenetic reprogramming. Nat Commun. 2011;2: 241 doi: 10.1038/ncomms1240 21407207 10.1038/ncomms1240

[26] Inoue A, Zhang Y. Replication-dependent loss of 5-hydroxymethylcytosine in mouse preimplantation embryos. Science. 2011;334: 194 doi: 10.1126/science.1212483 21940858 10.1126/science.1212483 PMC3799877

[27] Amouroux R, Nashun B, Shirane K, Nakagawa S, Hill PWS, D’Souza Z, et al supplement De novo DNA methylation drives 5hmC accumulation in mouse zygotes. Nat Cell Biol. 2016;18: 1–5. 26751286 10.1038/ncb3296 PMC4765106

[28] Li X, Ito M, Zhou F, Youngson N, Zuo X, Leder P, et al A maternal-zygotic effect gene, Zfp57, maintains both maternal and paternal imprints. Dev Cell. 2008;15: 547–557. doi: 10.1016/j.devcel.2008.08.014 18854139 10.1016/j.devcel.2008.08.014 PMC2593089

[29] Messerschmidt DM, de Vries W, Ito M, Solter D, Ferguson-Smith A, Knowles BB. Trim28 is required for epigenetic stability during mouse oocyte to embryo transition. Science. 2012;335: 1499–1502. doi: 10.1126/science.1216154 22442485 10.1126/science.1216154

[30] Nakamura T, Arai Y, Umehara H, Masuhara M, Kimura T, Taniguchi H, et al PGC7/Stella protects against DNA demethylation in early embryogenesis. Nat Cell Biol. 2007;9: 64–71. doi: 10.1038/ncb1519 17143267 10.1038/ncb1519

[31] Hirasawa R, Chiba H, Kaneda M, Tajima S, Li E, Jaenisch R, et al Maternal and zygotic Dnmt1 are necessary and sufficient for the maintenance of DNA methylation imprints during preimplantation development. Genes Dev. 2008;22: 1607–1616. doi: 10.1101/gad.1667008 18559477 10.1101/gad.1667008 PMC2428059

[32] Howell CY, Bestor TH, Ding F, Latham KE, Mertineit C, Trasler JM, et al Genomic imprinting disrupted by a maternal effect mutation in the Dnmt1 gene. Cell. 2001;104: 829–838. doi: 10.1016/S0092-8674(01)00280-X 11290321 10.1016/s0092-8674(01)00280-x

[33] Cirio MC, Ratnam S, Ding F, Reinhart B, Navara C, Chaillet JR. Preimplantation expression of the somatic form of Dnmt1 suggests a role in the inheritance of genomic imprints. BMC Dev Biol. 2008;8: 9 doi: 10.1186/1471-213X-8-9 18221528 10.1186/1471-213X-8-9 PMC2266903

[34] Unoki M, Nishidate T, Nakamura Y. ICBP90, an E2F-1 target, recruits HDAC1 and binds to methyl-CpG through its SRA domain. Oncogene. 2004;23: 7601–7610. doi: 10.1038/sj.onc.1208053 15361834 10.1038/sj.onc.1208053

[35] Rottach A, Frauer C, Pichler G, Bonapace IM, Spada F, Leonhardt H. The multi-domain protein Np95 connects DNA methylation and histone modification. Nucleic Acids Res. 2010;38: 1796–1804. doi: 10.1093/nar/gkp1152 20026581 10.1093/nar/gkp1152 PMC2847221

[36] Nady N, Lemak A, Walker JR, Avvakumov G V, Kareta MS, Achour M, et al Recognition of multivalent histone states associated with heterochromatin by UHRF1 protein. J Biol Chem. 2011;286: 24300–24311. doi: 10.1074/jbc.M111.234104 21489993 10.1074/jbc.M111.234104 PMC3129210

[37] Arita K, Ariyoshi M, Tochio H, Nakamura Y, Shirakawa M. Recognition of hemi-methylated DNA by the SRA protein UHRF1 by a base-flipping mechanism. Nature. 2008;455: 818–821. doi: 10.1038/nature07249 18772891 10.1038/nature07249

[38] Avvakumov G V, Walker JR, Xue S, Li Y, Duan S, Bronner C, et al Structural basis for recognition of hemi-methylated DNA by the SRA domain of human UHRF1. Nature. 2008;455: 822–825. doi: 10.1038/nature07273 18772889 10.1038/nature07273

[39] Hashimoto H, Horton JR, Zhang X, Bostick M, Jacobsen SE, Cheng X. The SRA domain of UHRF1 flips 5-methylcytosine out of the DNA helix. Nature. 2008;455: 826–829. doi: 10.1038/nature07280 18772888 10.1038/nature07280 PMC2602803

[40] Nishiyama A, Yamaguchi L, Sharif J, Johmura Y, Kawamura T, Nakanishi K, et al Uhrf1-dependent H3K23 ubiquitylation couples maintenance DNA methylation and replication. Nature. 2013;502: 249–253. doi: 10.1038/nature12488 24013172 10.1038/nature12488

[41] Qin W, Wolf P, Liu N, Link S, Smets M, Mastra F La, et al DNA methylation requires a DNMT1 ubiquitin interacting motif (UIM) and histone ubiquitination. Cell Res. 2015;25: 911–929. doi: 10.1038/cr.2015.72 26065575 10.1038/cr.2015.72 PMC4528052

[42] Karagianni P, Amazit L, Qin J, Wong J. ICBP90, a novel methyl K9 H3 binding protein linking protein ubiquitination with heterochromatin formation. Mol Cell Biol. 2008;28: 705–717. doi: 10.1128/MCB.01598-07 17967883 10.1128/MCB.01598-07 PMC2223417

[43] Arita K, Isogai S, Oda T, Unoki M, Sugita K, Sekiyama N, et al Recognition of modification status on a histone H3 tail by linked histone reader modules of the epigenetic regulator UHRF1. Proc Natl Acad Sci U S A. 2012;109: 12950–12955. doi: 10.1073/pnas.1203701109 22837395 10.1073/pnas.1203701109 PMC3420164

[44] Wang C, Shen J, Yang Z, Chen P, Zhao B, Hu W, et al Structural basis for site-specific reading of unmodified R2 of histone H3 tail by UHRF1 PHD finger. Cell Res. 2011;21: 1379–1382. doi: 10.1038/cr.2011.123 21808299 10.1038/cr.2011.123 PMC3193461

[45] Rajakumara E, Wang Z, Ma H, Hu L, Chen H, Lin Y, et al PHD finger recognition of unmodified histone H3R2 links UHRF1 to regulation of euchromatic gene expression. Mol Cell. 2011;43: 275–284. doi: 10.1016/j.molcel.2011.07.006 21777816 10.1016/j.molcel.2011.07.006 PMC4691841

[46] Deng Q, Ramskold D, Reinius B, Sandberg R. Single-cell RNA-seq reveals dynamic, random monoallelic gene expression in mammalian cells. Science. 2014;343: 193–196. doi: 10.1126/science.1245316 24408435 10.1126/science.1245316

[47] de Vries WN, Binns LT, Fancher KS, Dean J, Moore R, Kemler R, et al Expression of Cre recombinase in mouse oocytes: a means to study maternal effect genes. Genesis. 2000;26: 110–112. doi: 10.1002/(SICI)1526-968X(200002)26:2<110::AID-GENE2>3.0.CO;2–8 10686600

[48] Maquat LE. Nonsense-mediated mRNA decay: splicing, translation and mRNP dynamics. Nat Rev Mol Cell Biol. 2004;5: 89–99. doi: 10.1038/nrm1310 15040442 10.1038/nrm1310

[49] Smallwood SA, Tomizawa S, Krueger F, Ruf N, Carli N, Segonds-Pichon A, et al Dynamic CpG island methylation landscape in oocytes and preimplantation embryos. Nat Genet. 2011;43: 811–814. doi: 10.1038/ng.864 21706000 10.1038/ng.864 PMC3146050

[50] Wang L, Zhang J, Duan J, Gao X, Zhu W, Lu X, et al Programming and inheritance of parental DNA methylomes in mammals. Cell. 2014;157: 979–991. doi: 10.1016/j.cell.2014.04.017 24813617 10.1016/j.cell.2014.04.017 PMC4096154

[51] Miura F, Enomoto Y, Dairiki R, Ito T. Amplification-free whole-genome bisulfite sequencing by post-bisulfite adaptor tagging. Nucleic Acids Res. 2012;40: e136 doi: 10.1093/nar/gks454 22649061 10.1093/nar/gks454 PMC3458524

[52] Wagner EJ, Carpenter PB. Understanding the language of Lys36 methylation at histone H3. Nat Rev Mol Cell Biol. 2012;13: 115–126. doi: 10.1038/nrm3274 22266761 10.1038/nrm3274 PMC3969746

[53] Dahl JA, Jung I, Aanes H, Greggains GD, Manaf A, Lerdrup M, et al Broad histone H3K4me3 domains in mouse oocytes modulate maternal-to-zygotic transition. Nature. 2016;537: 548–552. doi: 10.1038/nature19360 27626377 10.1038/nature19360 PMC6283663

[54] Cardoso MC, Leonhardt H. DNA methyltransferase is actively retained in the cytoplasm during early development. J Cell Biol. 1999;147: 25–32. doi: 10.1083/jcb.147.1.25 10508852 10.1083/jcb.147.1.25 PMC2164986

[55] Liang C-C, Zhan B, Yoshikawa Y, Haas W, Gygi SP, Cohn MA. UHRF1 is a sensor for DNA interstrand crosslinks and recruits FANCD2 to initiate the Fanconi anemia pathway. Cell Rep. 2015;10: 1947–1956. doi: 10.1016/j.celrep.2015.02.053 25801034 10.1016/j.celrep.2015.02.053 PMC4386029

[56] Tian Y, Paramasivam M, Ghosal G, Chen D, Shen X, Huang Y, et al UHRF1 contributes to DNA damage repair as a lesion recognition factor and nuclease scaffold. Cell Rep. 2015;10: 1957–1966. doi: 10.1016/j.celrep.2015.03.038 25818288 10.1016/j.celrep.2015.03.038 PMC4748712

[57] Zhang H, Liu H, Chen Y, Yang X, Wang P, Liu T, et al A cell cycle-dependent BRCA1-UHRF1 cascade regulates DNA double-strand break repair pathway choice. Nat Commun. 2016;7: 10201 doi: 10.1038/ncomms10201 26727879 10.1038/ncomms10201 PMC4728409

[58] Quenneville S, Verde G, Corsinotti A, Kapopoulou A, Jakobsson J, Offner S, et al In embryonic stem cells, ZFP57/KAP1 recognize a methylated hexanucleotide to affect chromatin and DNA methylation of imprinting control regions. Mol Cell. 2011;44: 361–372. doi: 10.1016/j.molcel.2011.08.032 22055183 10.1016/j.molcel.2011.08.032 PMC3210328

[59] Hatanaka Y, Inoue K, Oikawa M, Kamimura S, Ogonuki N, Kodama EN, et al Histone chaperone CAF-1 mediates repressive histone modifications to protect preimplantation mouse embryos from endogenous retrotransposons. Proc Natl Acad Sci U S A. 2015;112: 14641–14646. doi: 10.1073/pnas.1512775112 26546670 10.1073/pnas.1512775112 PMC4664303

[60] Zhao Q, Zhang J, Chen R, Wang L, Li B, Cheng H, et al Dissecting the precise role of H3K9 methylation in crosstalk with DNA maintenance methylation in mammals. Nat Commun. 2016;7: 12464 doi: 10.1038/ncomms12464 27554592 10.1038/ncomms12464 PMC5426519

[61] Meilinger D, Fellinger K, Bultmann S, Rothbauer U, Bonapace IM, Klinkert WEF, et al Np95 interacts with de novo DNA methyltransferases, Dnmt3a and Dnmt3b, and mediates epigenetic silencing of the viral CMV promoter in embryonic stem cells. EMBO Rep. 2009;10: 1259–1264. doi: 10.1038/embor.2009.201 19798101 10.1038/embor.2009.201 PMC2756565

[62] Xu X, Li C, Garrett-Beal L, Larson D, Wynshaw-Boris A, Deng CX. Direct removal in the mouse of a floxed neo gene from a three-loxP conditional knockout allele by two novel approaches. Genesis. 2001;30: 1–6. doi: 10.1002/gene.1025 11353511 10.1002/gene.1025

[63] Jackson-Grusby L, Beard C, Possemato R, Tudor M, Fambrough D, Csankovszki G, et al Loss of genomic methylation causes p53-dependent apoptosis and epigenetic deregulation. Nat Genet. 2001;27: 31–39. doi: 10.1038/83730 11137995 10.1038/83730

[64] Kubo N, Toh H, Shirane K, Shirakawa T, Kobayashi H, Sato T, et al DNA methylation and gene expression dynamics during spermatogonial stem cell differentiation in the early postnatal mouse testis. BMC Genomics. 2015;16: 624 doi: 10.1186/s12864-015-1833-5 26290333 10.1186/s12864-015-1833-5 PMC4546090

[65] Toh H, Shirane K, Miura F, Kubo N, Ichiyanagi K, Hayashi K, et al Software updates in the Illumina HiSeq platform affect whole-genome bisulfite sequencing. BMC Genomics. 2017;18: 31 doi: 10.1186/s12864-016-3392-9 28056787 10.1186/s12864-016-3392-9 PMC5217569

[66] Krueger F, Andrews SR. Bismark: a flexible aligner and methylation caller for Bisulfite-Seq applications. Bioinformatics. 2011;27: 1571–1572. doi: 10.1093/bioinformatics/btr167 21493656 10.1093/bioinformatics/btr167 PMC3102221

[67] Speir ML, Zweig AS, Rosenbloom KR, Raney BJ, Paten B, Nejad P, et al The UCSC Genome Browser database: 2016 update. Nucleic Acids Res. 2016;44: D717–D725. doi: 10.1093/nar/gkv1275 26590259 10.1093/nar/gkv1275 PMC4702902

[68] Schultz MD, Schmitz RJ, Ecker JR. “Leveling” the playing field for analyses of single-base resolution DNA methylomes. Trends Genet. 2012;28: 583–585. doi: 10.1016/j.tig.2012.10.012 23131467 10.1016/j.tig.2012.10.012 PMC3523709

[69] Sasagawa Y, Nikaido I, Hayashi T, Danno H, Uno KD, Imai T, et al Quartz-Seq: a highly reproducible and sensitive single-cell RNA sequencing method, reveals non-genetic gene-expression heterogeneity. Genome Biol. 2013;14: R31 doi: 10.1186/gb-2013-14-4-r31 23594475 10.1186/gb-2013-14-4-r31 PMC4054835

[70] Kim D, Pertea G, Trapnell C, Pimentel H, Kelley R, Salzberg SL. TopHat2: accurate alignment of transcriptomes in the presence of insertions, deletions and gene fusions. Genome Biol. 2013;14: R36 doi: 10.1186/gb-2013-14-4-r36 23618408 10.1186/gb-2013-14-4-r36 PMC4053844

[71] Liao Y, Smyth GK, Shi W. FeatureCounts: An efficient general purpose program for assigning sequence reads to genomic features. Bioinformatics. 2014;30: 923–930. doi: 10.1093/bioinformatics/btt656 24227677 10.1093/bioinformatics/btt656

