# Supplementary material for: Role of UHRF1 in de novo DNA methylation in oocytes and maintenance methylation in preimplantation embryos
Source: PLoS Genet. 2017 Oct 4;13(10):e1007042. doi: 10.1371/journal.pgen.1007042 (PMC5643148; doi:10.1371/journal.pgen.1007042)
Supplement: S1 Table — (PDF) [file pgen.1007042.s008.pdf]

**S1 Table. Sequencing and mapping summary of WGBS.**

| Sample | Genotype | Sequenced reads | Uniquely mapped reads | Average depth per strand | Bisulfite conversion rate (%) |
|---|---|---|---|---|---|
| Blastocyst | | | | | |
| Control replicate 1 | *Uhrf1*$^{2lox/+}$ | 392,656,847 | 172,298,548 | 3.1 | 99.4 |
| Control replicate 2 | *Uhrf1*$^{2lox/+}$ | 158,397,135 | 71,529,414 | 1.7 | 99.5 |
| *Dnmt1* mat-KO replicate 1 | *Dnmt1*$^{1lox/+}$ | 530,041,385 | 279,767,131 | 5.2 | 99.5 |
| *Dnmt1* mat-KO replicate 2 | *Dnmt1*$^{1lox/+}$ | 144,454,789 | 66,905,125 | 1.5 | 99.5 |
| *Uhrf1* mat-KO replicate 1 | *Uhrf1*$^{1lox/+}$ | 408,763,226 | 190,098,421 | 3.4 | 99.4 |
| *Uhrf1* mat-KO replicate 2 | *Uhrf1*$^{1lox/+}$ | 298,618,108 | 141,335,924 | 3.3 | 99.5 |
| FGO | | | | | |
| Control replicate 1 | *Uhrf1*$^{2lox/2lox}$ | 607,390,746 | 339,829,529 | 6.3 | 99.5 |
| Control replicate 2[1)] | *Uhrf1*$^{+/+}$ | 695,199,202 | 383,311,717 | 7.8 | 99.6 |
| *Dnmt1* KO[1)] | *Dnmt1*$^{1lox/1lox}$ | 861,916,953 | 471,342,287 | 7.5 | 99.5 |
| *Uhrf1* KO replicate 1 | *Uhrf1*$^{1lox/1lox}$ | 561,695,524 | 321,494,573 | 6.0 | 99.5 |
| *Uhrf1* KO replicate 2 | *Uhrf1*$^{1lox/1lox}$ | 322,961,417 | 167,596,098 | 3.9 | 99.4 |

[1)]Shirane *et al*., PLoS Genet, 2013
