# Supplementary material for: Role of UHRF1 in de novo DNA methylation in oocytes and maintenance methylation in preimplantation embryos
Source: PLoS Genet. 2017 Oct 4;13(10):e1007042. doi: 10.1371/journal.pgen.1007042 (PMC5643148; doi:10.1371/journal.pgen.1007042)
Supplement: S2 Table — (PDF) [file pgen.1007042.s009.pdf]

S2 Table. Number of methylated cytosines.

Sample	Genotype	CG 5mC	CG Total C	CHG 5mC	CHG Total C	CHH 5mC	CHH Total C
FGO							
Control[1)]	*Uhrf1*$^{2lox/2lox\ or\ +/+}$	93,863,126	243,339,359	38,471,739	1,093,560,979	104,700,461	3,458,664,784
Dnmt1 KO[1)]	*Dnmt1*$^{1lox/1lox}$	54,479,097	151,510,932	35,152,007	807,735,328	94,863,903	2,579,198,801
Uhrf1 KO	*Uhrf1*$^{1lox/1lox}$	51,052,791	164,746,261	18,290,660	625,681,166	52,078,027	2,012,372,210
Blastocyst							
Control	*Uhrf1*$^{2lox/+}$	12,091,822	84,631,546	2,823,687	444,988,284	9,010,011	1,485,909,807
Dnmt1 mat-KO	*Dnmt1*$^{1lox/+}$	8,815,301	152,115,027	3,483,740	787,122,826	10,710,159	2,492,576,562
Uhrf1 mat-KO	*Uhrf1*$^{1lox/+}$	4,501,128	129,657,719	6,175,725	675,163,607	20,286,779	2,233,844,172

The numbers obtained from all reads in the WGBS replicates.

[1)]Shirane *et al*., PLoS Genet, 2013
