# Supplementary material for: Role of UHRF1 in de novo DNA methylation in oocytes and maintenance methylation in preimplantation embryos
Source: PLoS Genet. 2017 Oct 4;13(10):e1007042. doi: 10.1371/journal.pgen.1007042 (PMC5643148; doi:10.1371/journal.pgen.1007042)
Supplement: S3 Table — (PDF) [file pgen.1007042.s010.pdf]

**S3 Table. Criteria for Group 1–4 regions.**

| Group | CG methylation in control FGO | CG methylation difference between control and *Uhrf1* KO FGO | Number of 10-kb regions |
|---|---|---|---|
| 1 | 40 – 80% | <20% | 28,332 |
| 2 | 40 – 80% | ≥20% | 17,440 |
| 3 | ≥80% | <20% | 50,204 |
| 4 | ≥80% | ≥20% | 4,175 |
