# Supplementary material for: Role of UHRF1 in de novo DNA methylation in oocytes and maintenance methylation in preimplantation embryos
Source: PLoS Genet. 2017 Oct 4;13(10):e1007042. doi: 10.1371/journal.pgen.1007042 (PMC5643148; doi:10.1371/journal.pgen.1007042)
Supplement: S4 Table — (PDF) [file pgen.1007042.s011.pdf]

S4 Table. Sequencing and mapping summary of RNA-seq.

Sample	Genotype	Sequenced reads	Mapped reads	Mapping efficiency (%)
Control FGO replicate 1	*Uhrf1*$^{2lox/2lox}$	27,186,289	25,996,907	97.6
Control FGO replicate 2	*Uhrf1*$^{2lox/2lox}$	4,763,938	4,306,868	92.1
Uhrf1 KO FGO replicate 1	*Uhrf1*$^{1lox/1lox}$	25,382,196	24,195,528	97.4
Uhrf1 KO FGO replicate 2	*Uhrf1*$^{1lox/1lox}$	5,088,083	4,527,834	91.0
