# Supplementary material for: Role of UHRF1 in de novo DNA methylation in oocytes and maintenance methylation in preimplantation embryos
Source: PLoS Genet. 2017 Oct 4;13(10):e1007042. doi: 10.1371/journal.pgen.1007042 (PMC5643148; doi:10.1371/journal.pgen.1007042)
Supplement: S5 Table — (PDF) [file pgen.1007042.s012.pdf]

S5 Table. List of PCR primers.

Primer name	Sequence
**Genotyping**	
Uhrf1 exon 3F	5’-AAGACTGTGTGGGAGGACAC-3’
Uhrf1 intron 3R	5’-AAGTGATGTGGTCACAGAGG-3’
Uhrf1 exon 6R	5’-TGTTGAGCTGAGAGTCATTC-3’
Dnmt1 exon 3F	5’-AGCCAGTTGTGTGACTTGG-3’
Dnmt1 exon 4R	5’-CTTGGGCCTGGATCTTGGGGA-3’
Dnmt1 intron 5R	5’-ATGCATAGGAACAGATGTGTGC-3’
Cre F	5’-GCAGAACCTGAAGATGTTCGCGAT-3’
Cre R	5’-AGGTATCTCTGACCAGAGTCATCC-3’
**RT- PCR**	
Dnmt3a exon 9F	5’-GCCGAATTGTGTCTTGGTGGATGACA-3’
Dnmt3a exon 10R	5’-CCTGGTGGAATGCACTGCAGAAGGA-3’
Dnmt3b exon 3F	5’-TTCAGTGACCAGTCCTCAGACACGAA-3’
Dnmt3b exon 4R	5’-TCAGAAGGCTGGAGACCTCCCTCTT-3’
Dnmt3L exon 10F	5’-GTGCGGGTACTGAGCCTTTTTAGA-3’
Dnmt3L exon 11R	5’-CGACATTTGTGACATCTTCCACGTA-3’
Dnmt1 exon7 Fwd	5’-CCTAGTTCCGTGGCTACGAGGAGAA-3’
Dnmt1 exon8 Rev	5’-TCTCTCTCCTCTGCAGCCGACTCA-3’
Uhrf1 qEx2F (exon 2)	5’-CGTGAACTCTCTGTCCAGGT-3’
Uhrf1 qEx3R (exon 3)	5’-GTCATTGAGGCGCACATCAT-3’
Uhrf1 F2 (exon 12)	5’-GCATCTACAAGGTGGTGAAG-3’
Uhrf1 R2 (exon 13)	5’-AGGCTCTGTGTCATCTCGTC-3’
Gapdh F1	5’-ATGTTTGTGATGGGTGTGAAC-3’
Gapdh R1	5’-CATTGTCATACGGAAATGAGC-3’
